# Effective, Long-Term, Neutrophil Depletion Using a Murinized Anti-Ly-6G 1A8 Antibody

**DOI:** 10.3390/cells11213406

**Published:** 2022-10-27

**Authors:** Patricia A. Olofsen, Marjolein C. Stip, J. H. Marco Jansen, Chilam Chan, Maaike Nederend, Ralph G. Tieland, Maria Tsioumpekou, Jeanette H. W. Leusen

**Affiliations:** Center for Translational Immunology, University Medical Center Utrecht, 3584 CX Utrecht, The Netherlands

**Keywords:** neutrophils, neutrophil depletion, in vivo neutrophil targeting, immunotherapy, cancer

## Abstract

Neutrophils are crucial innate immune cells but also play key roles in various diseases, such as cancer, where they can perform both pro- and anti-tumorigenic functions. To study the function of neutrophils in vivo, these cells are often depleted using Ly-6G or Gr-1 depleting antibodies or genetic “knockout” models. However, these methods have several limitations, being only partially effective, effective for a short term, and lacking specificity or the ability to conditionally deplete neutrophils. Here, we describe the use of a novel murinized Ly-6G (1A8) antibody. The murinized Ly-6G antibody is of the mouse IgG2a isotype, which is the only isotype that can bind all murine Fcγ receptors and C1q and is, therefore, able to activate antibody-dependent cellular cytotoxicity (ADCC), antibody-dependent phagocytosis (ADCP) and complement-dependent cytotoxicity (CDC) pathways. We show that this mouse-Ly-6G antibody shows efficient, long-term, and near-complete (>90%) neutrophil depletion in the peripheral blood of C57Bl6/J, Balb/c, NXG and SCID mice for up to at least four weeks, using a standardized neutrophil depletion strategy. In addition, we show that neutrophils are efficiently depleted in the blood and tumor tissue of IMR32 tumor-bearing SCID mice, analyzed six weeks after the start of the treatment.

## 1. Introduction

Neutrophils are the most abundant immune cells in the human body, comprising 50–70% of the white blood cell population in the circulation. They are essential for innate immune reactions but also play key roles in tumorigenesis, where they can perform both pro- and anti-tumorigenic functions [1,2,3,4]. To study the in vivo function of neutrophils, Ly-6G (clone 1A8) or Gr-1 (clone RB6-8C5) depleting antibodies are frequently used. Over the years, it has become clear that both these antibodies show efficacy problems, with depletion being only partially effective, transient, or lacking specificity [5,6,7]. The misinterpretation of results is observed in many studies due to the usage of the same antibody clone for both depletion and detection, thereby blocking the epitope for staining, resulting in false-negative results. In addition, whereas Ly-6G is only present on neutrophils, Ly-6C, the other receptor recognized by the Gr-1 antibody, can also be found on subsets of monocytes, macrophages, dendritic cells, and lymphocytes [8,9,10]. Besides the differences in target specificity, Ly-6G and Gr-1 antibodies differ in isotype and efficacy. While the Gr-1 antibody is a rat IgG2b, described to work via the fast-acting complement-dependent cytotoxicity (CDC) pathway [11,12], Ly-6G is a rat IgG2a antibody, shown to mediate neutrophil killing through “slow” Fc-dependent antibody-dependent cellular cytotoxicity (ADCC) and antibody-dependent phagocytosis (ADCP) by monocytes and macrophages, making the neutrophil depletion less efficient [13]. Also, since both depletion antibodies are of rat origin, mouse anti-rat antibodies produced by the mice play a role in reduced efficacy, where increased clearance of the injected depletion antibodies is observed after one week of treatment [14].

In recent years, various suggestions have been made to overcome some of the problems observed with Ly-6G and Gr-1 depletion antibodies. Boivin et al. described a double antibody-based depletion strategy using Ly-6G and an additional anti-rat IgG2a antibody to facilitate an “isotype switch,” resulting in a 90% decrease in blood neutrophil numbers [14]. Others have tried to generate genetic neutrophil “knockout” models by targeting various key components of neutrophils (e.g., Csf3r, Cxcr2, Gfi-1), resulting in only partial (60–90%) depletion of mature neutrophil subsets [15,16,17,18]. In addition to being partially effective, the majority of these genetic models are constitutive, and neutrophils can only be added to the equation by performing bone marrow transplantations.

Here we describe the use of a murinized version of the Ly-6G 1A8 antibody (hereafter referred to as mouse-Ly-6G), which has the same variable region of the fragment antigen-binding (Fab) domain as the original rat-Ly-6G 1A8 antibody but is of the mouse IgG2a isotype. Mouse IgG2a is the only isotype that can bind all murine Fcγ receptors, where it binds with high affinity to the activating FcγRI and FcγRIV receptors and has low affinity for FcγRIII and the inhibitory FcγRIIb receptor, making it the ideal isotype to induce ADCC and ADCP [19]. In addition, IgG2a can efficiently bind C1q, thereby activating the complement pathway and inducing CDC [20]. Due to the design of the mouse-Ly-6G antibody, it has the potential to show high specificity (since Ly-6G is only present on neutrophils), high efficacy (it should be able to activate ADCC, ADCP, and CDC), and be able to work more long-term (no mouse anti-rat antibodies will be produced, preventing clearance of the injected antibody). Here we describe an optimized and standardized neutrophil depletion strategy and show that the mouse-Ly-6G antibody shows efficient, long-term, and near-complete (>90%), neutrophil depletion in the peripheral blood of C57Bl6/J, Balb/c, NXG and SCID mice for up to four weeks. In addition, we show that neutrophils can be efficiently depleted in the blood and tumor tissue of IMR32 tumor bearing SCID mice, even six weeks after the start of the treatment.

## 2. Materials and Methods

### 2.1. Mice

Mice were maintained in the animal facility of the University of Utrecht. Experiments were conducted using C57Bl/6J (C57Bl/6JRj), Balb/c (Balb/cByJRj), NXG (NOD.Prkdc^scid^ Il2rg^tm1^/Rj), SCID (NOD.CB17-Prkdc^scid/scid^/Rj), and human FcαRI (CD89) transgenic SCID mice (all housed and bred at Janvier Labs, Paris, France) [21], or C57Bl/6J FcRγ^−/−^ (C57Bl/6JRj FcRγ-chain knockout) mice (housed and bred at the University of Utrecht) [22]. Mice were housed in groups under a 12:12 light–dark cycle, with food and water available ad libitum. Mice were randomized based on age, and both treatment and analysis were performed blind. Upon transfer to Utrecht, mice were acclimatized for at least 1 week prior to the start of the experiment.

All experiments were performed in accordance with international guidelines and approved by the National Central Authority for Scientific Procedures on Animals (CCD) and the local experimental animal welfare body (AVD115002016410).

### 2.2. Neutrophil Depletion

Mice were injected intra-peritoneally (i.p.) with 25, 50, or 100 μg mouse-Ly-6G, 50 or 100 μg rat- Ly-6G, or solvent control (PBS) 3 times a week. The rat-Ly-6G (1A8) hybridoma has been sequenced by Absolute Antibody and transformed into a commercially available recombinant mouse antibody (see Table 1 for more information on the depletion antibodies used). To evaluate neutrophil depletion, blood was drawn via cheek puncture before antibody injection and collected in Lithium-Heparin tubes (Sarstedt, #20.1345, Etten-Leur, the Netherlands). Blood was stored on ice to prevent internalization of CD115, a marker used for flow cytometry detection of monocytes.

### 2.3. Flow Cytometry

Myeloid cell composition and neutrophil depletion were assessed using the antibodies used in Table 2. To detect other leukocytes present in the blood, we used the antibodies shown in Table 3. For blood and tumor samples from IMR32 tumor-bearing mice, the antibodies depicted in Table 4 were used. Additionally, TO-PRO-3 (Thermo Fisher, #10710194, Bleiswijk, the Netherlands) was added to tumor samples in a 1:50000 dilution to exclude dead cells. 

Antibodies were diluted in FACS buffer (PBS + 0.1%BSA + 0.1% sodium azide) and added to mouse blood in a 1:1 ratio (15 μL each). After 30–45-min incubation at 4 °C, 1 mL 1× FACS lysis solution (BD, #349202, 10× diluted in mQ) was added to wash away unbound antibodies and lyse the erythrocytes. Cells were lysed for 5–8 min at room temperature, followed by a centrifugation step (1800 rpm, 5 min, 4 °C). Cells were washed in 1 mL FACS buffer, centrifuged (1800 rpm, 5 min, 4 °C), and resuspended in 150 μL FACS buffer containing microsphere latex beads (Thermo Fisher, #11550696, Bleiswijk, the Netherlands, 3000×). For tumor samples, 2.5 × 10^6^ tumor cells were stained in a 50 µL antibody mix for 30–45-min at 4 °C. After washing, cells were fixed in 4% paraformaldehyde.

Flow cytometry on blood samples was performed using a BD Canto II flow cytometer, while a BD LSR Fortessa was used to measure tumor samples. Data analysis was done using FlowJo (TreeStar, Ashland, OR, USA).

### 2.4. Neutrophil Attraction Model in IMR32 Tumor Bearing Mice

SCID mice were injected subcutaneously (s.c.) with 2.5 × 10^6^ IMR32 cells in a 1:2 mix of IMR32 cells in PBS and Vitrogel Hydrogel Matrix (Tebu Bio, #306VHM01, Heerhugowaard, the Netherlands). Tumor outgrowth was measured using a caliper (length × width × height). Twenty-eight days after the start of the experiment, mice were randomized into treatment groups based on tumor size and age, after which mice were treated i.p. with solvent control (PBS) or IgA ch14.18 antibodies in combination with a IgG1 PGLALA SIRPα-D1 fusion protein (produced as described before) to attract intratumoral neutrophils [23,24,25]. PBS and 25 mg/kg IgA ch14.18 antibodies were administered 3 times a week, while the SIRPα-D1 fusion protein was given every 9 days at a dose of 30 mg/kg. To induce neutrophil depletion, mice were treated with 100 μg mouse-Ly-6G 3 times a week. Blood was sampled at days 35, 42, 49, 56, 63, and 70 after tumor cell injection (days 7, 14, 21, 28, 35, and 42 after the start of the treatments) as described above and analyzed by flow cytometry.

Neutrophil infiltration in the tumor microenvironment was evaluated on day 70. Mouse tumors were carefully excised and collected in ice-cold PBS. Tumors were cut smaller and digested using the mouse tumor dissociation kit from Miltenyi (#130-096-730, Leiden, the Netherlands). Up to 1 g of tumor tissue was transferred to C tubes (Miltenyi, #130-096-334, Leiden, the Netherlands) containing enzyme mix (RPMI culture medium, 100 μL Enzyme D, 50 μL Enzyme R, and 10μL Enzyme A) and the 37C_m_TDK_1 program was run on a gentleMACS Octo Dissociator. After dissociation, cells were put through a 70 µm cell strainer in medium containing 10% FCS (Sigma), washed with FACS buffer, and stained for flow cytometry.

### 2.5. Quantification and Statistical Analysis

Data are presented as mean ± SEM. Comparison of multiple groups was performed using one-way ANOVA with Bonferroni correction, repeated measures one-way ANOVA with Bonferroni correction, or two-way ANOVA with Bonferroni correction. Statistical analyses were performed using GraphPad Prism 9.3.0 (GraphPad Software Inc., San Diego, CA, USA). A *p*-value < 0.05 was considered significant.

## 3. Results

### 3.1. Mouse-Ly-6G Depletes Neutrophils More Efficiently Than Rat-Ly-6G in C57Bl6/J Mice

**Figure 1:**
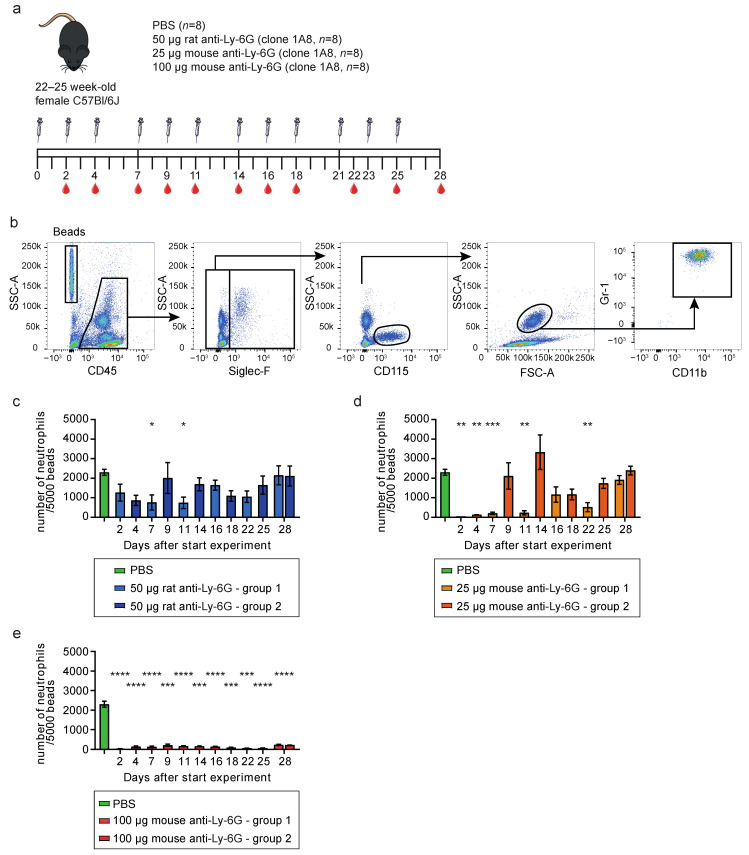
Mouse-Ly-6G efficiently depletes neutrophils in old C57Bl6/J mice. (**a**) Experimental set-up; i.p. injections with the depletion-antibodies or vehicle control (PBS) were performed three times a week for four weeks. Blood was drawn via cheek puncture before injection with the antibodies; (**b**) FACS dot plots of a representative PBS-treated mouse, showing the gating strategy to retrieve the number of neutrophils per 5000 beads. First, the latex beads and CD45^+^ cells were selected. From the CD45^+^ population, Siglec-F and CD115 positive cells were excluded. Based on SSC/FSC profile, the neutrophils were selected and purity was confirmed using Gr-1 and CD11b positivity; (**c–e**) Longitudinal analysis of the number of CD45^+^ Siglec-F^−^ CD115^−^ SSC^high^ Gr-1^+^ CD11b^+^ neutrophils per 5000 beads in the peripheral blood (*n* = four mice per subgroup) showing (**c**) inefficient depletion with 50 μg rat-Ly-6G antibody; (**d**) efficient short-term depletion with 25 μg mouse-Ly-6G spanning the first week, and (**e**) efficient long-term depletion with 100 μg mouse-Ly-6G. Data is presented as mean with standard error of the mean (SEM). Statistics: one-way ANOVA with Bonferroni correction, * = *p* < 0.05, ** = *p* < 0.01, *** = *p* < 0.001, **** = *p* < 0.0001.

To investigate whether the mouse-Ly-6G antibody was able to efficiently deplete neutrophils, we decided to use old (>20 weeks) C57Bl6/J mice since these mice show high neutrophil-turnover and are the most refractory to rat-Ly-6G mediated neutrophil depletion [14]. 22–25 week-old mice were injected three times a week with two different concentrations (25 or 100 μg) of the mouse-Ly-6G antibody, 50 μg rat-Ly-6G 1A8 antibody, or solvent control (PBS) (Figure 1a). The eight mice per group were randomly divided into two subgroups to be able to draw blood three times a week (four mice per time point) and subsequently perform flow cytometric analysis of the leukocyte composition. First, hematopoietic cells were selected using CD45, followed by the exclusion of Siglec-F positive eosinophils and CD115 positive monocytes. To detect remaining neutrophils, we used the Gr-1 RB6-8C5 antibody clone (to prevent possible epitope masking of Ly-6G) in combination with CD11b staining and the specific forward and sideward scatter properties of neutrophils (Figure 1b). Analysis of the number of neutrophils per 5000 microsphere latex beads indicated that the rat-Ly-6G antibody did not result in efficient neutrophil depletion at most of the time points analyzed (Figure 1c and Figure S1b), as was described previously [5,7,14]. In contrast, the lowest concentration of mouse-Ly-6G (25 μg) significantly depleted neutrophils at days 2, 4, 7, 11, and 22 after the start of the experiment, indicating that 25 μg mouse-Ly-6G can efficiently deplete neutrophils for up to one week, after which it is insufficient to compete with neutrophil production (Figure 1d and Figure S1c). Increasing the concentration of the mouse-Ly-6G antibody to 100 μg per mouse resulted in an almost complete absence of neutrophils in the peripheral blood at all time points tested, indicating efficient long-term depletion up to four weeks (Figure 1e and Figure S1d,e).

### 3.2. 100μg Mouse-Ly-6G Efficiently Depletes Neutrophils in C57Bl6/J, Balb/c, NXG, and SCID Mice

The promising results in C57Bl6/J mice tempted us to investigate whether neutrophils could also be depleted in other mouse strains, e.g., Balb/c, NXG, and SCID. Where C57Bl6/J and Balb/c mice are immunocompetent, SCID and NXG mice are immunodeficient. SCID mice depict a B- and T-cell deficiency, and NXG mice lack mature B-, T-, and NK-cells, show defective dendritic cells and macrophages due to impaired IL2R signaling, and lack hemolytic complement because of a 2-base pair deletion in the C5 structural gene [26,27].

**Figure 2:**
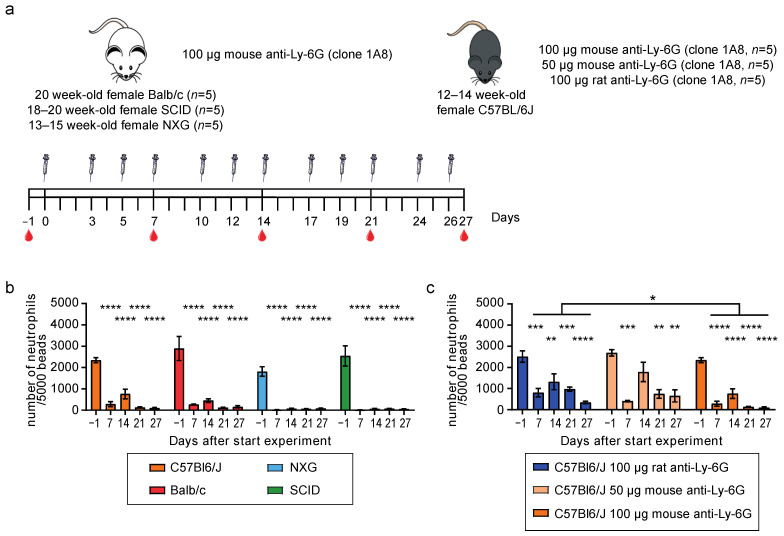
Mouse-Ly-6G efficiently depleted neutrophils in C57Bl6/J, Balb/c, NXG, and SCID mice. (**a**) Experimental set-up; i.p. injections with the depletion antibodies were performed three times a week for four weeks. Blood was drawn via cheek puncture once a week before injection with the antibodies; (**b**,**c**) Longitudinal analysis of the number of CD45^+^ Siglec-F^−^ CD115^−^ SSC^high^ Gr-1^+^ CD11b^+^ neutrophils in the peripheral blood (*n* = five mice per group) per 5000 latex beads, showing; (**b**) Significant, almost complete, neutrophil depletion in all mouse strains tested (C57Bl6/J, Balb/c, NXG, and SCID) upon treatment with 100 μg mouse-Ly-6G antibody, and (**c**) Significantly better neutrophil depletion in 100 μg mouse-Ly-6G treated C57Bl6/J mice than when treated with 100 μg rat-Ly-6G antibody. Data is presented as mean with SEM. Statistics: one-way ANOVA with Bonferroni correction. Repeated measures one-way ANOVA with Bonferroni correction was used to compare groups in Figure 2c, * = *p* < 0.05, ** = *p* < 0.01, *** = *p* < 0.001, **** = *p* < 0.0001.

Mice (*n* = 5 per strain) were injected three times a week with 100 μg mouse-Ly-6G antibody, and blood was analyzed once per week (Figure 2a). Flow cytometry analysis showed significant and almost complete depletion of neutrophils in all mouse strains, even the immunodeficient NXG mice that could not deplete antibodies via CDC (Figure 2b and Figure S2a–e).

To investigate whether the mouse-Ly-6G antibody was indeed outperforming the rat-Ly-6G antibody, we injected C57Bl6/J mice with 50 μg mouse-Ly-6G (compare to Figure 1c), 100 μg mouse-Ly-6G or 100 μg rat-Ly-6G. As shown in Figure 2c, 100 μg rat-Ly-6G injected mice showed significantly reduced numbers of neutrophils in the peripheral blood. However, the reduction was only partial and significantly less (*p* = 0.04) than when the same concentration of mouse-Ly-6G antibody was used. Of note, these mice were 12–14 weeks old, thereby ten weeks younger than the mice used in Figure 1, depicting lower neutrophil turnover, explaining why these mice were not completely refractory to rat-Ly-6G treatment [14]. Injecting mice with 50 μg mouse-Ly-6G did reduce the number of neutrophils in the peripheral blood, but the reduction was incomplete after one week, indicating that 100 μg mouse-Ly-6G is needed to efficiently deplete the excess neutrophils produced upon neutropenia.

### 3.3. Neutrophils Can Efficiently Be Depleted Intratumorally in IMR32 Tumor Bearing Mice

**Figure 3:**
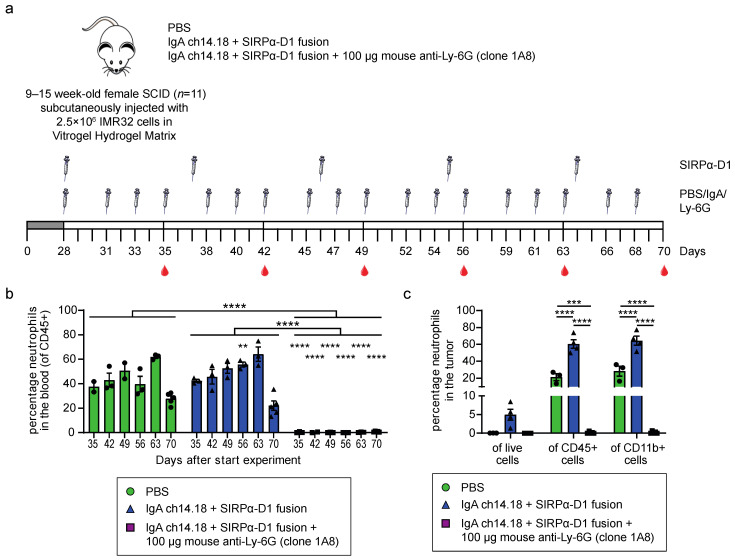
Mouse-Ly-6G efficiently depletes neutrophils in the blood and tumor of IMR32 tumor-bearing SCID mice (**a**) Experimental set-up; 2.5 × 10^6^ IMR32 cells were s.c. injected in SCID mice in a 1:2 mix with Vitrogel Hydrogel Matrix. Tumors were established for 28 days, after which mice were randomized over the different treatment groups, and treatment was started. I.p. injections with PBS, IgA ch14.18, and mouse-Ly-6G were performed three times a week for six weeks. The SIRPα-D1 fusion protein was injected i.p. every nine days. Blood was drawn via cheek puncture once a week before injection with the antibodies; (**b**) Longitudinal analysis of the percentage of SSC^high^ Ly-6C^low^ Ly-6G^+^ CD11b^+^ neutrophils in the peripheral blood (*n* = 2–5 mice per group) of the CD45+ leukocyte population, showing complete neutrophil depletion upon treatment with 100 μg mouse-Ly-6G antibody. Of note; blood at day 70 was collected from the orbit and processed in a similar fashion as the tumor material, meaning cells were fixed at a later time point, possibly resulting in some neutrophil cell death; (**c**) Intratumoral analysis of the percentage of SSC^high^ Ly-6C^low^ Ly-6G^+^ CD11b^+^ neutrophils of the live (TO-PRO-3 negative) cells, CD45+ leukocytes, and CD11b+ myeloid populations. Data showed increased tumor infiltration when the mice were treated with IgA ch14.18/SIRPα-D1 fusion, which was completely ablated when 100 μg mouse Ly-6G was added. Data is presented as mean with SEM. Statistics: one-way ANOVA with Bonferroni correction. Repeated measures one-way ANOVA with Bonferroni correction was used to compare groups in Figure 3b, ** = *p* < 0.01, *** = *p* < 0.001, **** = *p* < 0.0001.

Multiple studies highlight the inefficiency of intratumoral neutrophil depletion using the rat-Ly-6G antibody [6,28]. To investigate whether the mouse-Ly-6G antibody is capable of efficient intratumoral neutrophil depletion, we injected SCID mice s.c. with 2.5 × 10^6^ IMR32 cells in a 1:2 mix with Vitrogel Hydrogel Matrix. To attract neutrophils to the tumor site, mice were treated with a combination of IgA ch14.18 antibodies and an IgG1 PGLALA SIRPα-D1 fusion protein [29]. Other treatment groups consisted of vehicle control (PBS) and IgA/SIRPα-D1 in combination with 100 μg mouse-Ly-6G (Figure 3a). Flow cytometric analysis of the blood showed complete neutrophil depletion in the IgA/SIRPα-D1/mouse-Ly-6G treated mice until the end of the experiment, six weeks after the start of the treatment (Figure 3b). As expected, flow cytometric analysis of the tumor indicated a significant increase in the number of neutrophils when mice were treated with IgA/SIRPα-D1 (Figure 3c). Some neutrophils could be detected in the tumors of PBS-treated mice. However, in the IgA/SIRPα-D1/mouse-Ly-6G treated mice, neutrophils were completely absent, indicating that both the neutrophils attracted by the IgA/SIRPα-D1 treatment were ablated, as well as the neutrophils normally present in the tumors (Figure 3c).

### 3.4. Neutrophil Depletion with Mouse-Ly-6G Is Not Solely Complement- or Fc Receptor-Mediated

**Figure 4:**
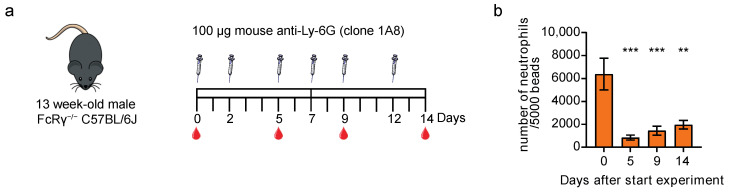
Mouse-Ly-6G efficiently depletes neutrophils in FcRγ-deficient mice. (**a**) Experimental set-up; FcRγ^−/−^ mice (*n* = 5) were i.p. injected three times a week with 100 μg mouse-Ly-6G antibody, and blood was drawn via cheek puncture before the start of the experiment, and at days 5, 9, and 14; (**b**): FcRγ^−/−^ mice show depletion of the number of CD45^+^ Siglec-F^−^ CD115^−^ SSC^high^ Gr-1^+^ CD11b^+^ neutrophils in the peripheral blood (shown per 5000 latex beads) at all time points. Data is presented as mean with SEM. Statistics: one-way ANOVA with Bonferroni correction, ** = *p* < 0.01, *** = *p* < 0.001.

Neutrophil depletion with the rat-Ly-6G antibody is shown to be dependent on mononuclear phagocytosis but is facilitated by complement [30]. Since neutrophils were also efficiently depleted in NXG mice (Figure 2b), which, among other defects, did not show complement activity, the mouse-Ly-6G antibody should have been able to exert its function independent of complement [27]. Studies using allogeneic, species-matched antibodies concluded that antibody-mediated cell depletion is complement-independent and mediated by monocytes and macrophages through the cooperation of multiple Fc receptors [31,32,33]. Hinting towards a role of these cells in neutrophil depletion was the observation that some of the 100 μg mouse-Ly-6G treated animals showed increased numbers of CD115^+^ monocytes (both Ly-6C positive and negative subsets) in the bloodstream, while this was not observed in rat-Ly-6G treated mice or with other leukocyte subsets (Figure S3a,b). To investigate whether mouse-Ly-6G antibody-mediated cell depletion was mediated through the cooperation of multiple Fc receptors, we used gamma chain “knockout” mice (C57Bl/6J FcRγ^−/−^; Figure 4a), which lack the Fc receptor gamma chain essential for, e.g., normal Fc receptor signaling, ADCC, and ADCP [22,34]. Treating FcRγ^−/−^ mice with 100 μg mouse-Ly-6G resulted in a significant and almost complete depletion of neutrophils (Figure 4b), indicating that ADCC/ADCP was not the only mechanism of action. In line with this, the design of the mouse-Ly-6G, using an IgG2a isotype, suggests that this antibody could activate both ADCC/ADCP and CDC pathways, thereby possibly explaining why both NXG mice (no CDC) and FcRγ^−/−^ mice (no ADCC and ADCP) showed efficient neutrophil depletion upon mouse-Ly-6G treatment, emphasizing the increased possibilities while using this antibody [19,20].

## 4. Discussion

In this study, we evaluated the efficacy and specificity of the murinized Ly-6G 1A8 antibody as a tool to efficiently deplete neutrophils. By injecting C57Bl6/J, Balb/c, NXG, and SCID mice three times a week i.p., with 100 μg of the mouse-Ly-6G antibody, we showed efficient (>90%) neutrophil depletion for up to at least four weeks, while IMR32-tumor-bearing SCID mice showed neutrophil depletion for up to six weeks. The mouse-Ly-6G outperformed the originally described rat-Ly-6G antibody and showed almost complete neutrophil depletion even in old C57Bl6/J mice, resistant to rat-Ly-6G antibody treatment [14].

Problems with the efficacy of the rat-Ly-6G antibody have clouded the results of many studies. Epitope masking by the injected depletion antibody has resulted in numerous false-negative results due to the usage of the same antibody clone for staining. In addition, the neutropenia achieved by using Ly-6G-depletion antibodies resulted in increased differentiation of myeloid progenitors present in the bone marrow, thereby producing more mature neutrophils and releasing them into the bloodstream [35]. The majority of cells that were present after Ly-6G treatment were shown to be newly made neutrophils, indicating that rat-Ly-6G depletion was not able to compensate for the increased neutrophil production [14]. In addition, the newly released neutrophils express lower levels of Ly-6G, making it more difficult to deplete and detect them [14]. Here, we describe a method to efficiently detect neutrophils by flow cytometry, not solely relying on Ly-6G expression. By excluding Siglec-F positive eosinophils and CD115 positive monocytes, neutrophils could be easily detected by their high sideward scatter (SSC) properties. Confirmation of the neutrophil phenotype with Gr-1 and CD11b expression shows >95% purity.

The Gr-1 antibody RB6-8C5 has shown fewer efficacy problems due to recognizing more antigens on the surface of neutrophils (Ly-6G and Ly-6C) while also being a rat IgG2a antibody functioning via the fast-acting CDC pathway, achieving ~90% reduction of peripheral blood neutrophils [14]. However, Gr-1 also targets Ly-6C positive subsets of monocytes, macrophages, dendritic cells, and lymphocytes, making it impossible, although often done, to draw conclusions about the role of neutrophils alone [8,9,10]. With the mouse-Ly-6G antibody, the best of both worlds was combined since depletion efficacy mimicked the Gr-1 RB6-8C5 antibody, while only Ly-6G-positive neutrophils were depleted, keeping the other leukocyte populations intact. In addition, by virtue of being a mouse IgG2a antibody, the mouse-Ly-6G can activate ADCC, ADCP, and CDC, therefore also being effective in models lacking one of these pathways. Furthermore, as a mouse antibody, the production of anti-rat antibodies was prevented, circumventing the problem of reduced efficacy due to increased clearance of the injected depletion antibodies. Due to this, it was possible to efficiently deplete neutrophils for at least four weeks using the same treatment regimen and emphasizing the importance of antibody species and isotypes.

A reliable, transient, long-term neutrophil depletion model is essential to study various functions of neutrophils, e.g., their role in tumor development and progression. In addition, neutrophils have been shown to play major roles in autoimmune diseases and IgA-mediated immunotherapy [36,37,38,39]. To really grasp the role neutrophils play in these diseases and therapies, it is important to be able to efficiently and reproducibly deplete neutrophils for the complete duration of the experiment. Using the treatment regimen described above, it is possible to efficiently deplete neutrophils in the peripheral blood for at least four weeks in C57Bl6/J, Balb/c, NXG, and SCID mice. IMR32 tumor-bearing SCID mice showed efficient neutrophil depletion in blood and tumor tissue six weeks after the start of the treatment and ten weeks after the injection of the IMR32 tumor cells, making it possible to, e.g., study the role of neutrophils in tumor outgrowth, even after the tumor cells have engrafted. Therefore, the mouse-Ly-6G antibody is the perfect tool to conditionally, efficiently, and specifically deplete neutrophils in blood and tissue for a long period of time.

## Figures and Tables

**Table 1:** Antibodies used to deplete neutrophils.

Antigen	Species	Isotype	Company	Catalog Number
Ly-6G (clone 1A8)	Mouse	IgG2a Kappa	Absolute Antibody	Ab00295-2.0
Ly-6G (clone 1A8)	Rat	IgG2a Kappa	Bio X Cell	BP0075-1_5 mg

**Table 2:** Antibodies used to assess myeloid cell composition.

Antigen	Fluorophore	Company	Catalog Number	Dilution
CD11b	Alexa488	BD	557672	100×
GR-1 (clone RB6-8C5)	PE-Cy7	eBioscience	25-5931-82	200×
CD115	APC	Sony Biotechnology	1277550	200×
Ly-6C	APC-Cy7	Biolegend	128025	200×
Siglec-F/CD170	BV421	BD	562681	100×
CD45	BV510	Biolegend	103137	200×

**Table 3:** Antibodies used to detect other leukocytes.

Antigen	Fluorophore	Company	Catalog Number	Dilution
CD3e	PE	Biolegend	100308	200×
NK1.1	PerCP-Cy5.5	Biolegend	108727	100×
B220/CD45R	PE-Cy7	BD	561881	100×
CD11b	APC	eBioscience	17-0112-80	100×
Ly-6C	APC-Cy7	Biolegend	128025	200×
CD45	Pacific Blue	Biolegend	103126	200×
GR-1 (clone RB6-8C5)	Pacific Orange	Invitrogen	RM3030	100×

**Table 4:** Antibodies used to analyze blood and tumor samples of IMR32 tumor-bearing mice.

Antigen	Fluorophore	Company	Catalog Number	Dilution
CD11b	FITC	Pharmingen	553310	50×
Ly-6C	PerCP-Cy5.5	Biolegend	128012	100×
Ly-6G	PE-Cy7	Biolegend	127618	300×
Siglec-F/CD170	BV421	BD	562681	100×
CD45	BV510	Biolegend	103137	200×

## Data Availability

Not applicable.

## Supplementary Materials

The following supporting information can be downloaded at: www.mdpi.com/article/10.3390/cells11213406/s1, Figure S1: Analysis of the number of neutrophils upon antibody treatment (related to Figure 1); Figure S2: Gating strategy to retrieve the number of neutrophils per 5000 beads (related to Figure 2); Figure S3: Number of monocytes, eosinophils, T-, B-, and NK-cells upon Ly-6G treatment (related to Figure 1).
