# Supplementary material for: Effective, Long-Term, Neutrophil Depletion Using a Murinized Anti-Ly-6G 1A8 Antibody
Source: Cells. 2022 Oct 27;11(21):3406. doi: 10.3390/cells11213406 (PMC9656769; doi:10.3390/cells11213406)
Supplement: Supplementary file 1 [file cells-11-03406-s001.zip › cells-1971636-supplementary.pdf]

Supplemental Figures

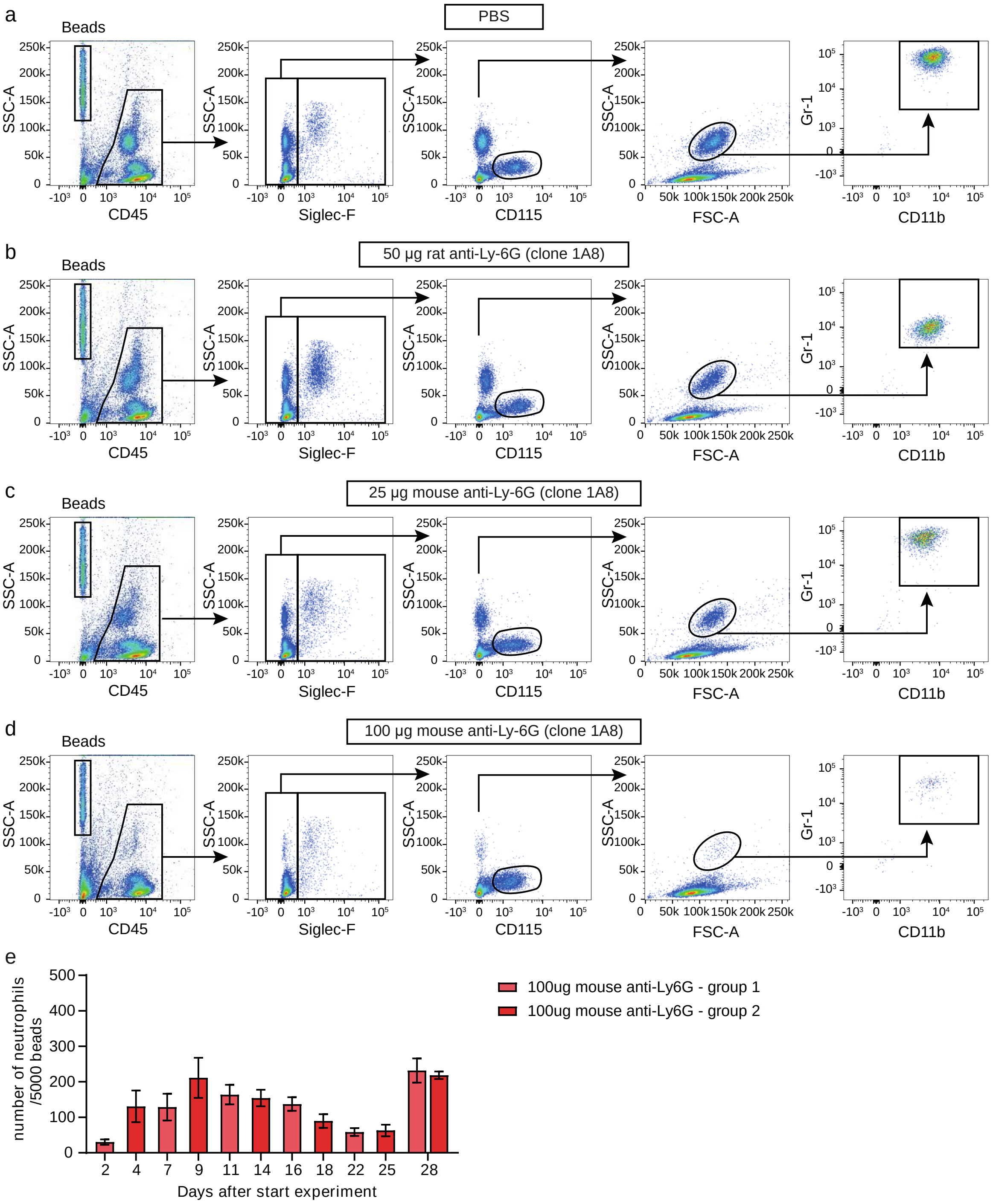

Figure S1: Analysis of the number of neutrophils upon antibody treatment (related to Figure 1). (**a-e**): FACS dot plots showing representative blood samples at day 28 after the start of the experiment for (**a**) PBS injected control mice, (**b**) 50 µg rat-Ly-6G injected mice, (**c**) 25 µg mouse-Ly-6G injected mice, and (**d**) 100 µg mouse-Ly-6G injected mice. (**e**) Zoom in of the longitudinal analysis of the number of $CD45^{+}$ Siglec-F^{-} $CD115^{-}$ SSC^{high} Gr-1^{+} $CD11b^{+}$ neutrophils per 5000 beads in the peripheral blood (n=4 mice per subgroup) of 100 µg mouse-Ly-6G treated mice, as depicted in Figure 1e.

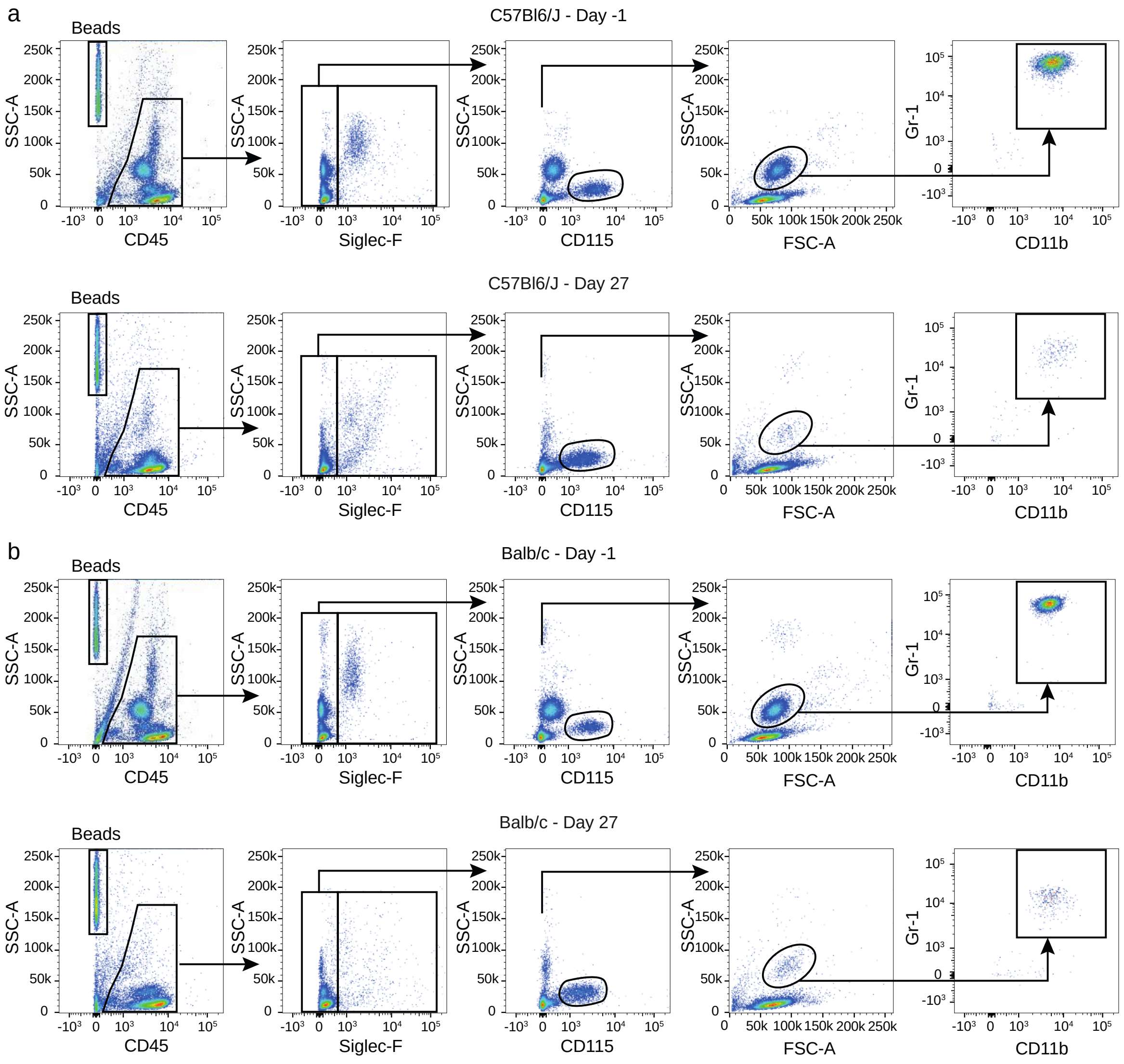
a
C57Bl6/J - Day -1
C57Bl6/J - Day 27
b
Balb/c - Day -1
Balb/c - Day 27
Beads
SSC-A
CD45
Siglec-F
CD115
FSC-A
Gr-1
CD11b

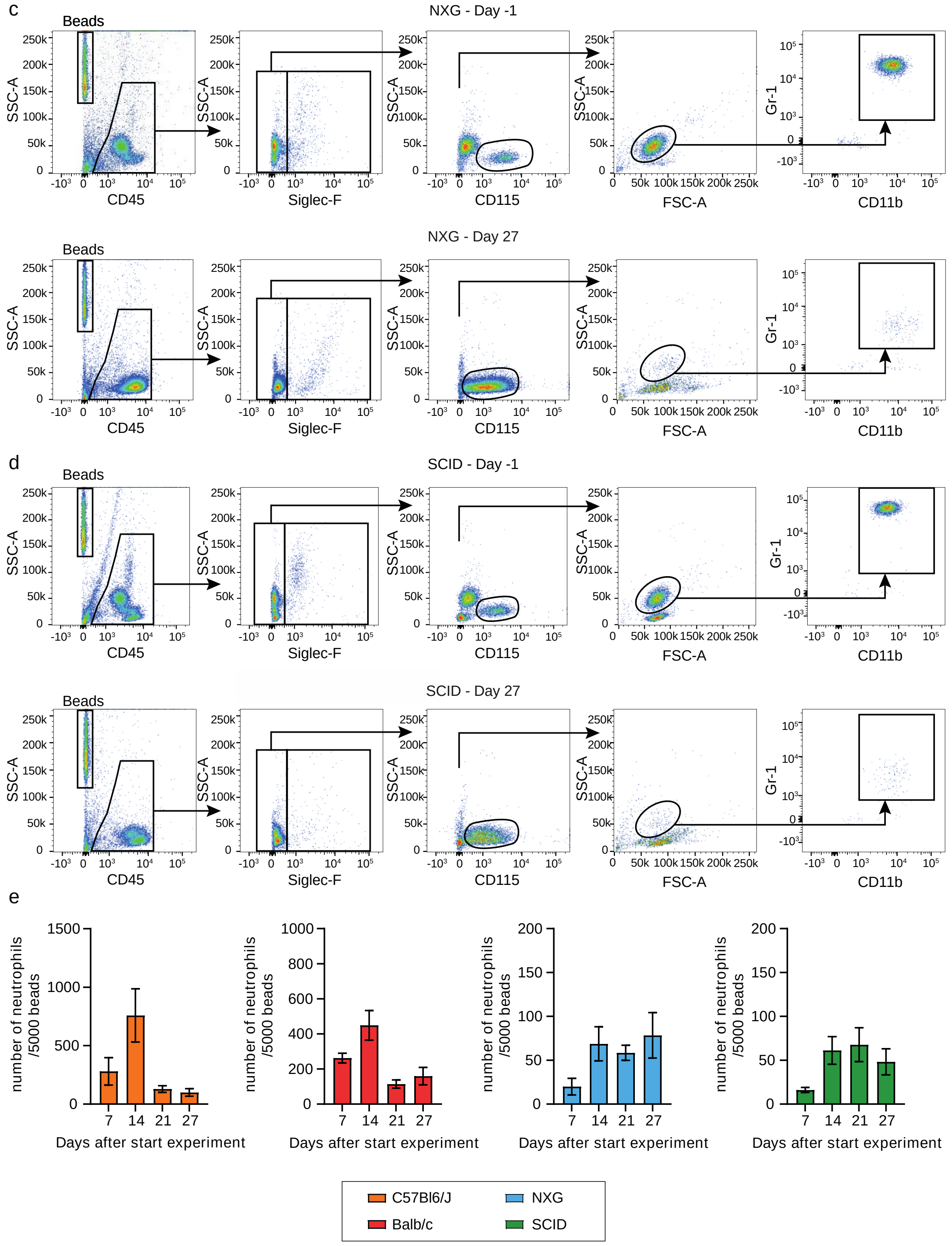

Figure S2: Gating strategy to retrieve the number of neutrophils per 5000 beads (related to Figure 2). (**a-d**): FACS dot plots showing representative blood samples at the day before the start of the experiment (day -1) and at the end of the experiment (day 27) for (**a**) C57Bl6/J, (**b**) Balb/c, (**c**) NXG, and (**d**) SCID mice, injected with 100 µg mouse-Ly-6G. (**e**) Zoom in of the longitudinal analysis of the number of CD45^{+} Siglec-F^{-} CD115^{-} SSC^{high} Gr-1^{+} CD11b^{+} neutrophils per 5000 beads in the peripheral blood (n=5 mice per group) of 100 µg mouse-Ly-6G treated mice, as depicted in Figure 2b.

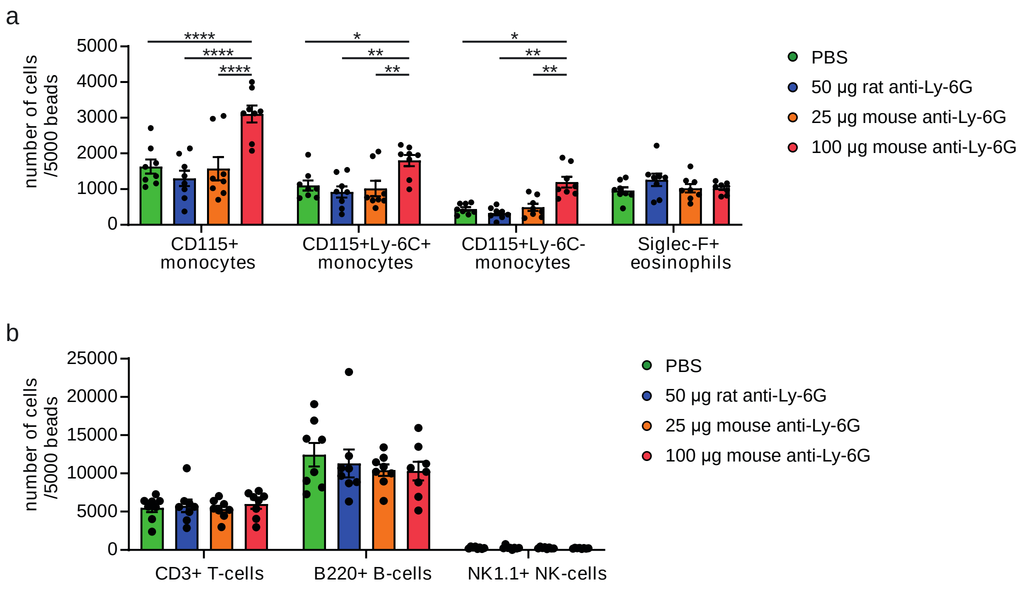

Figure S3: Number of monocytes, eosinophils, T-, B-, and NK-cells upon Ly6g treatment (related to Figure 1). Flow cytometry analysis of the number of leukocytes in the peripheral blood at day 28 after the start of the experiment (n=8 mice per group, as shown in Figure 1) per 5000 latex beads, showing (**a**): a significant increase in both Ly-6C^{+} and Ly-6C^{-} CD115^{+} monocytes, but not eosinophils, upon neutrophil depletion with 100 µg mouse-Ly-6G antibody, and (**b**): no differences in CD3^{+} T-cells, B220^{+} B-cells and NK1.1^{+} NK-cells. Statistics: two-way ANOVA with Bonferroni correction, *=p<0.05, **=p<0.01, ***=p<0.001, ****=p<0.0001.
